# Monetary incentives have only limited effects on auditory distraction: evidence for the automaticity of cross-modal attention capture

**DOI:** 10.1007/s00426-020-01455-5

**Published:** 2020-12-19

**Authors:** Raoul Bell, Laura Mieth, Axel Buchner, Jan Philipp Röer

**Affiliations:** 1 Department of Experimental Psychology, Heinrich Heine University Düsseldorf, 40225 Düsseldorf, Germany; 2 Department of Psychology and Psychotherapy, Witten/Herdecke University, Witten, Germany

## Abstract

The duplex-mechanism account of auditory distraction postulates that two distinct forms of auditory distraction can be distinguished by whether or not they can be cognitively controlled. While the interference-by-process component of auditory distraction is postulated to be automatic and independent of cognitive control, the stimulus-aspecific attention capture by auditory deviants and the stimulus-specific attentional diversion by auditorily presented distractor sentences should be suppressed by increased task engagement. Here we test whether incentive-induced changes in task engagement affect the disruption of serial recall by auditory deviants (Experiment 1) and distractor sentences (Experiment 2). Monetary incentives substantially affected recall performance in both experiments. However, the incentive-induced changes in task engagement had only limited effects on auditory distraction. In Experiment 2, increased task engagement was associated with a small decrease of distraction relative to a quiet condition, but strong effects of auditory distraction on performance persisted in conditions of high task engagement in both experiments. Most importantly, and in contrast to the predictions of the duplex-mechanism account, the effects of stimulus-aspecific attention capture (Experiment 1) and stimulus-specific attentional diversion (Experiment 2) remained unaffected by incentive-induced changes in task engagement. These findings are consistent with an automatic-capture account according to which only the processes responsible for the deliberate memorization of the target items are dependent on controlled mental effort while the attention capture by auditory deviants and the attentional diversion by distractor speech are largely automatic.

Cognitive performance is often found to be disrupted by auditory distraction (e.g., Ellermeier & Zimmer, 2014). Most of the evidence comes from laboratory studies in which participants are instructed to focus on visual targets and to ignore auditory distractors but do not receive any external incentives for following these instructions. In a strict sense, these standard experimental tasks constitute model tasks for real-life situations in which a deviation from ongoing processing does not have serious consequences (such as when listening to music while reading a book for pleasure). It is unclear whether the results obtained in such tasks generalize to situations with external incentives for good performance because direct evidence regarding the effect of external incentives on auditory distraction is rare (for an exception, see Ball et al., 2018). This lack of knowledge is unfortunate from an applied perspective as it is of great practical interest to know whether providing external incentives can be effectively used to counter the detrimental effects of auditory distraction in real-world settings where cognitive performance is critical but must be maintained under conditions of auditory distraction (e.g., in working or learning environments). Testing whether auditory distraction is modulated by external incentives is equally interesting from a theoretical perspective as accounts of auditory distraction make strong predictions about the influence of top-down cognitive control on auditory attention capture but the available evidence is mixed (e.g., Parmentier, 2014).

According to the expected value of control theory (Shenhav et al., 2013, 2016), people engage in cognitive control to maximize reward. Within this theory, exerting cognitive control is effortful and thus ascribed a subjective cost. The degree to which people are willing to invest mental effort in completing a task is determined by a cost–benefit analysis in which the expected payoffs are weighted against this cost. This implies that the degree to which people engage in cognitive control should be determined by the value of its outcome. Providing monetary incentives for putting effort into a demanding task can be expected to increase task engagement because the financial payoffs directly affect this cost–benefit analysis.

Accordingly, providing monetary incentives for good performance has been found to cause an increase of activity in brain regions associated with cognitive control that was accompanied by enhanced working-memory performance (Pochon et al., 2002; Taylor et al., 2004). Morey et al. (2011) used monetary incentives to examine selective resource-sharing in a dual-task working-memory paradigm involving a visual-spatial and an auditory task. They found evidence of an incentive-induced tradeoff between the two tasks—when the incentive for one of the tasks increased, performance in that task increased at the cost of a performance decrement in the other task, suggesting a high degree of flexibility in allocating attentional resources to the contents of working memory. When dual-task instructions allow for a flexible allocation of attentional resources, monetary incentives can thus affect the tradeoff between competing contents of working memory.

However, the allocation of attention is not always under cognitive control. In selective-attention paradigms, manipulating task engagement by providing external (monetary) incentives serves to test whether the allocation of attention is determined by goal-driven (controlled) or stimulus-driven (automatic) processes (Bucker & Theeuwes, 2014). For instance, there is evidence that the distracting effect of emotional (e.g., erotic) stimuli is reduced by providing external (monetary) incentives for focusing on simultaneously presented visual targets (Walsh et al., 2018). This finding can be interpreted as suggesting that orienting to intrinsically interesting stimuli is partly under cognitive control, implying that the tradeoff between the nominally relevant and irrelevant material involves a deliberate decision on how intensely each stream of information is to be processed. Other components of visual attention such as the initial orienting towards the abrupt onset of peripheral cues, by contrast, have been found to be unaffected by incentive-induced variations in task engagement, suggesting that these processes are stimulus-driven and escape cognitive control (Bucker & Theeuwes, 2014).

Here we examine the effects of monetary incentives on cross-modal selective attention and thereby test whether auditory distraction is under cognitive control. For this purpose, we selected the well-established irrelevant-sound paradigm (for reviews of the literature, see Banbury et al., 2001; Ellermeier & Zimmer, 2014). In this paradigm, a list of targets (e.g., digits) is visually presented and has to be serially recalled either immediately or after a short retention interval. During encoding and memorization, auditory distractors have to be ignored. The general disruption of serial recall by auditory distractors relative to a quiet condition is known as the *irrelevant-sound effect* (Ellermeier & Zimmer, 2014). The disruptive effect of distractor speech is particularly strong when complex naturalistic distractor material such as sentential speech is used (Bell et al., 2017; Hughes & Marsh, 2020). A subcomponent of the irrelevant-sound effect is the *changing-state effect* (Campbell et al., 2002; Jones et al., 1993). The changing-state effect refers to the observation that changing-state sequences consisting of different sounds (e.g., “A B C D E F G”) disrupt performance more than steady-state sequences consisting of repeated sounds (e.g., “A A A A A A A”). A changing-state effect has been observed with spoken letters (Campbell et al., 2002), digits (Tremblay & Jones, 1999), and one-syllable words as distractor material (LeCompte et al., 1997). The changing-state effect can also be elicited by non-speech sounds such as tone sequences (Jones & Macken, 1993), melodies (Schlittmeier et al., 2008), and instrumental sounds (Bell et al., 2019c). The changing-state effect can be distinguished from the *auditory-deviant effect* (Hughes et al., 2005; Lange, 2005). The auditory-deviant effect refers to the observation that deviations from ongoing auditory stimulation capture attention and disrupt performance. In most studies (Körner et al., 2017; Sörqvist, 2010; Vachon et al., 2017), an auditory steady-state sequence that is disrupted by a single deviating distractor sound (e.g., “A A A A B A A”) is compared to a regular steady-state sequence (e.g., “A A A A A A A”). However, an auditory-deviant effect can also be obtained with repeated distractors when the repetitions are unexpected (e.g., “A B C D D F G”). It is, therefore, the violation of an expectation rather than the auditory change per se that is responsible for the disruption (Hughes et al., 2007; Marsh et al., 2014; Röer et al., 2014).

According to the *duplex-mechanism account* (Hughes, 2014), the changing-state effect and the auditory-deviant effect represent two fundamentally different types of auditory distraction. The changing-state effect is viewed as an unavoidable side effect of the automatic seriation of the auditory input. When changes are detected, the auditory stream is pre-attentively segmented into auditory objects. The order of these objects is obligatorily processed. This unpreventable processing of order information interferes with the order processing underlying the serial rehearsal of the visual targets. The automatic processing of the auditory distractors is assumed to be unaffected by top-down cognitive control (Hughes, 2014). The duplex-mechanism account thus implies that the changing-state effect should remain unaffected by incentive-induced changes in task engagement. The predictions differ for disruptive effects that are attentional in nature. According to the duplex-mechanism account, there are two classes of attentional diversion. *Stimulus-aspecific attentional diversion* occurs when the auditory input deviates from an expected pattern. The auditory-deviant effect is the prototype of this class. The second class of attentional diversion occurs when the specific content of the distractor attracts attention. *Stimulus-specific attentional diversion* is exemplified in the disruptive effect of sentential speech that is partly attributed to the inherent interest of its meaning to those trying to ignore it (Hughes & Marsh, 2020). Importantly, according to the duplex-mechanism account the disengagement of focal attention is avoided in states of high task engagement (Hughes et al., 2013). The duplex-mechanism account, therefore, implies that increased task engagement reduces the disruptive effects of sequences containing deviant information and sentential speech relative to that of more simple distractor sequences consisting of one-syllable words.

To test these predictions, most previous studies have relied on manipulations of perceptual task difficulty. Hughes et al. (2013) reported that increasing the perceptual difficulty of the encoding of the visual targets abolished the auditory-deviant effect but did not affect the changing-state effect. Specifically, when the to-be-remembered targets were difficult to perceive because they were embedded in static visual noise, performance in the baseline condition was as good as when the to-be-remembered targets were easy to perceive, but auditory deviants had no measurable effect on performance anymore. The increased perceptual task demands were said to have caused an up-regulation of controlled, effortful processing aimed at compensating the decrease in task performance due to the perceptual degradation of the target items. This implies that the postulated effect of perceptual encoding difficulty on task engagement cannot be directly observed in baseline task performance because it is assumed to be just balanced out by the postulated negative effect of encoding difficulty on performance. Only the postulated effects on auditory distraction can be empirically validated. Importantly, within the duplex-mechanism account, disruption by auditory deviants depends on a tradeoff in the controlled allocation of attention and is therefore abolished by increased task engagement. The changing-state effect, by contrast, is postulated to be rooted in automatic processes that are not amenable to cognitive control and thus should remain unaffected by changes in task engagement (Hughes, 2014). Following up on this idea, Marsh et al. (2020) required their participants to serially recall hierarchical Navon figures in which small letters, when grouped together, formed the shapes of large letters. Marsh et al. reported that requiring participants to attend to the small letters abolished the auditory-deviant effect but had no effect on baseline performance or the changing-state effect. Provided that people have a bias to focus on large rather than on small letters, the requirement to overcome this tendency may signal an increased need for cognitive control, which may then decrease the controlled allocation of attention to auditory deviants but fail to influence the automatic seriation of changing-state speech. Stimulus-specific attentional diversion (exemplified by the disruptive power of distractors with emotional content relative to that of distractors without emotional content) was also found to be reduced under conditions of increased encoding difficulty (Marsh et al., 2018).

The predictions of the duplex-mechanism account can be contrasted with those of the automatic-capture account. According to this account, attention is exogenously captured by distractors against an individual’s best effort to concentrate on the task, which implies that the degree to which individuals have control over attention capture is severely limited, if it is possible at all (Körner et al., 2017; Röer et al., 2017). In contrast to the duplex-mechanism account, the automatic-capture account implies that both the changing-state effect and the auditory-deviant effect are rooted in the automatic processing of the auditory input, which leaves only the processes that underlie the memorization of the target items as being dependent on cognitive control. Therefore, any manipulation that induces an up-regulation of task engagement should have a global effect on the effortful processes underlying the voluntary (controlled) memorization of the target items but should have little effect on the automatic processing of changing-state and deviant distractors. These assumptions lead to the prediction that serial-recall performance should globally improve as a function of the up-regulation of task engagement, but the size of both the changing-state effect and the auditory-deviant effect should remain unaffected. This is, of course, only true under the possibly simplifying assumption that the quantitative or qualitative changes induced in the effortful processing of the target items have no bearing on auditory distraction. Fortunately, it has been reported that, if anything, the changing-state effect is more sensitive to the encoding and retention processes required by the primary task than the auditory-deviant effect (Vachon et al., 2017). If incentive-induced quantitative or qualitative changes in encoding and memorization affect auditory distraction at all, they can be expected to have a stronger influence on the changing-state effect than on the auditory-deviant effect, in direct contrast to the predictions of the duplex-mechanism account spelled out above. This allows for a clear empirical test of the two competing accounts.

In the oddball paradigm, evidence regarding the potential influence of cognitive control on disruption by auditory deviants is mixed (Parmentier, 2014). In a study by Parmentier and colleagues (2008), participants were required to perform a visual odd–even categorization task in which standard tones (presented in 90% of the trials) or novel environmental sounds (presented in 10% of the trials) had to be ignored. The same manipulation of perceptual encoding difficulty that was used by Hughes and colleagues (2013) had no effect on auditory distraction by novel environmental sounds that deviated from the standard tones. Mixed results were also obtained with regard to electrophysiological correlates of attention capture by auditory deviants. A recent meta-analysis found evidence suggesting that visual task difficulty reduces the mismatch negativity in response to deviant tones, but also revealed that the results may be compromised by a publication bias (Wiens et al., 2016). Furthermore, a preregistered follow-up study found that the electrophysiological correlates of the processing of auditory deviants remained unaffected by manipulations of visual attention (Wiens et al., 2019). The evidence that is available from these lines of research is thus not fully conclusive but tends to indicate that cognitive control over auditory distraction is limited. By contrast, evidence in the serial-recall paradigm—in which distraction is measured in terms of memory errors—at this point favors the view that distraction by auditory deviants is subject to cognitive control and can be abolished by increased task engagement (Hughes et al., 2013; Marsh et al., 2018, 2020). The present study, however, will challenge this view.

The main aim of the present study was to test the prediction of the duplex-mechanism account (Hughes, 2014) that changes in top-down task engagement should affect stimulus-aspecific attention capture and stimulus-specific attentional diversion while it should have no influence on the changing-state effect. The perceptual-degradation manipulation used in most previous studies (Hughes et al., 2013; Marsh et al., 2018) can be seen as an indirect manipulation because it primarily affects perceptual task difficulty, which is then assumed to cause a compensatory increase in task engagement. This assumed increase in task engagement is not reflected in baseline performance (without distraction) because it is overshadowed by the detrimental effects of perceptual task difficulty on performance. This introduces unnecessary uncertainty in the interpretation of the findings because perceptual degradation may have other effects on processing in addition to, or instead of, increasing task engagement. For instance, any effects on distraction may be mediated by bottom-up rather than top-down effects of attentional selection (Lavie et al., 2004). In contrast, the monetary-incentive manipulation used here allows to manipulate top-down task engagement without affecting the perceptual appearance of the to-be-remembered target stimuli. Monetary incentives can be expected to directly affect the cost–benefit analysis in which potential payoffs are weighted against the cost of exerting mental effort (Shenhav et al., 2013; 2016). The effectiveness of this manipulation can be directly tested by analyzing baseline task performance. If the manipulation is effective, performance in the control conditions must be better when good performance is monetarily rewarded relative to when it is not. Other than the authors of previous studies (Hughes et al., 2013; Marsh et al., 2018, 2020), we thus do not have to rely on the to-be-tested effect on auditory distraction as the only criterion for judging the effectiveness of the task-engagement manipulation.

As a critical test of the competing accounts, we will rely on the interaction between incentive condition (no incentive, monetary incentive) and auditory distraction. In Experiment 1, we focus on stimulus-aspecific attentional diversion. Provided that monetary incentives are effective in modulating task engagement, the duplex-mechanism account allows to derive the hypotheses that (1) the auditory-deviant effect should be abolished or substantially reduced when performance is incentivized relative to when it is not while (2) the changing-state effect should remain unaffected. By contrast, the automatic-capture account implies that both the auditory-deviant effect and the changing-state effect should remain unaffected by changes in task engagement. In Experiment 2, we focus on stimulus-specific attentional diversion. Specifically, we test whether the effect of sentential speech relative to a steady-state control condition is affected by monetary incentives. The duplex-mechanism account partly attributes the greater disruptive power of sentential speech to attentional diversion (Hughes & Marsh, 2020). The account, therefore, implies that (3) the increased disruptive effect of sentential speech relative to that of a sequence of repeated one-syllable words should be modulated by changes in task engagement. Both accounts discussed here thus imply parallel predictions for stimulus-specific and stimulus-aspecific attentional diversion: The duplex-mechanism account implies that the distraction caused by attentional diversion is suppressed or eliminated by enhanced task engagement while the automatic-capture account implies that attentional diversion remains unaffected by changes in task engagement.

The results of these empirical tests are not only theoretically relevant, but interesting from an applied perspective as well: They allow to determine whether auditory distraction can be modulated by the presence or absence of salient external incentives for good task performance. If such a modulation is robustly obtained, external incentives could be applied to reduce the problem of auditory distraction in (work or education) settings where optimal cognitive performance is critical but auditory distraction is unavoidable.

## Experiment 1

### Method

#### Participants

We aimed at recruiting at least 100 participants, but continued data collection until the end of the week in which we reached this aim. After one week, we had collected valid data sets of 113 participants (90 of whom were female). Four data sets had to be excluded prior to analysis (two because two students had participated twice, one because of a computer malfunction, and one because the participant had turned the volume of the computer down). Given *α * = 0.05 and the assumption that the population correlation of the differences between the no-incentive and the monetary-incentive condition among the levels of the distractor condition variable is ρ = 0.5, a total sample size of *N* = 113 allowed us to detect an interaction between the incentive and the distractor condition variables of η_p_^2^ = 0.12 with a statistical power of 1 – β = 0.95. The participants were recruited on campus of Heinrich Heine University Düsseldorf. Their age ranged from 18 to 40 years with a mean age of 23 (*SD* = 4) years. All participants received course credit or a monetary compensation of 4 € for participating. In addition, they could earn up to 5.76 € depending on their performance in the serial-recall task (see below).

#### Design, materials and procedure

A 2 × 3 design was used with incentive (no incentive, monetary incentive) and distractor condition (steady state, auditory deviant, changing state) as repeated-measures variables and serial recall as the dependent variable.

Participants were tested in sessions with up to 5 participants. They were seated in separate cubicles constructed with sound-absorbing walls and wore headphones with high-insulation hearing protection covers (beyerdynamic DT-150) plugged into the Apple iMac computer controlling the experiment.

Participants received standardized written instructions in which they were asked to concentrate only on the digits and to ignore the auditory modality. They were informed that the words heard through headphones were completely irrelevant for the task and would not become relevant later in the experiment.

The absence or presence of monetary incentives was manipulated within participants in a trial-based fashion. To familiarize participants with the procedure, the experiment started with 16 steady-state training trials, half of which were incentivized and half of which were not. Then the participants had to complete 16 steady-state trials, 16 auditory-deviant trials, and 16 changing-state trials. In half of the trials of each condition, performance was incentivized. Each participant completed the training and experimental trials in a different, randomly determined order.

Participants started each trial by pressing the space bar of the computer keyboard. Before each trial, they received a visual cue informing them about whether good performance would be rewarded. In the monetary-incentive condition, participants saw a € symbol in the middle of the screen for five seconds. In the no-incentive condition, a € symbol struck through by a red line was displayed. Participants knew that they would receive 2 cents for each digit they would recall at the correct serial position in each incentivized trial and that they would receive the total amount of money accumulated across all rewarded trials at the end of the experiment on top of what they would receive as a compensation for their participation. They were encouraged to try to collect as many cents as possible to receive as much money as possible. They were also informed that they would receive no additional money for their performance in non-incentivized trials.

One second after the € symbol had disappeared, a sequence of randomly ordered digits was presented visually. Each to-be-remembered sequence consisted of nine digits drawn from the set {1, 2, …, 9} without replacement. The digits were presented for 1 s each in black 80 pt Monaco font against a white background in the middle of the screen of the computer that controlled the experiment. Immediately after the presentation of the to-be-remembered digit sequence, nine question marks appeared in the middle of the screen which had to be replaced, one after another, with the remembered digits. The numbers were typed into the number pad of the keyboard of the computer that controlled the experiment. Participants were not allowed to correct or skip responses.

As soon as the € symbol disappeared, the auditory distractor sequence was played. The distractor sequences consisted of one-syllable German words drawn from the set {Berg [mountain; bɛʁk], Chef [boss; ʃɛf], Dank [thank; daŋk], Gold [gold; gɔlt], Haut [skin; haʊ̯t], Hof [yard; hoːf], Mund [mouth; mʊnt], Rand [edge; ʀant], Ruf [call; ʀuːf], Typ [type; tyːp], Wind [wind; vɪnt], Zeug [stuff; ʦɔɪ̯k]} (English translation and German pronunciation in brackets). The words were spoken by a female voice, recorded with a 44.1 sampling rate in 16 bit format, edited to last for 750 ms, normalized, and played at about 65 dB(A) Leq. For each steady-state sequence, one of the words was randomly drawn from this set and repeated 12 times. The auditory-deviant sequences were identical to the steady-state sequences, except that in each sequence one word was replaced by a different, randomly determined deviant word. The deviant word occurred randomly at positions 7, 8, 9, or 10 in the auditory distractor sequence. Each changing-state sequence consisted of the 12 distractor words played in a random order.

After each trial, participants received a summary feedback about their performance. In a monetary-incentive trial, participants were informed about the number of correctly recalled digits, their monetary reward resulting from remembering these digits, and the total number of cents earned so far during the experiment. In the no-incentive trials, participants received the same feedback but they always gained *0 cents* regardless of the number of digits they had recalled in the trial.

The experiment lasted about 28 min. The study did not involve deception as the bonus participants received at the end of the experiment did indeed depend on their performance in the incentivized trials. On average, participants earned 4.02 € in addition to their regular participation fee.

#### Data analysis

In line with previous studies (e.g., Bell et al., 2019a, b, c), a strict serial-recall criterion was used for scoring the data, which means that only items recalled at the correct serial position were scored as correct. We used the MANOVA approach to repeated-measures analyses (O'Brien & Kaiser, 1985). In our applications, all multivariate test criteria correspond to the same (exact) *F* statistic which is reported, and the Pillai-Bartlett *V* is equivalent to the partial eta squared (η_p_^2^) which is used as an effect-size measure (Bredenkamp & Erdfelder, 1985).

## Results

A 2 × 3 repeated-measures analysis with incentive and distractor condition as independent variables revealed a significant main effect of distractor condition, *F*(2,111) = 36.44, *p* < 0.001, η_p_^2^ = 0.40. In addition, serial recall was better when good performance was incentivized than when it was not, *F*(1,112) = 72.51, *p* < 0.001, η_p_^2^ = 0.39. Nevertheless, there was no interaction between these variables, suggesting that the effects of auditory distraction were not affected by monetary incentives, *F*(2,111) = 2.36, *p* = 0.10, η_p_^2^ = 0.04.

**Fig. 1:**
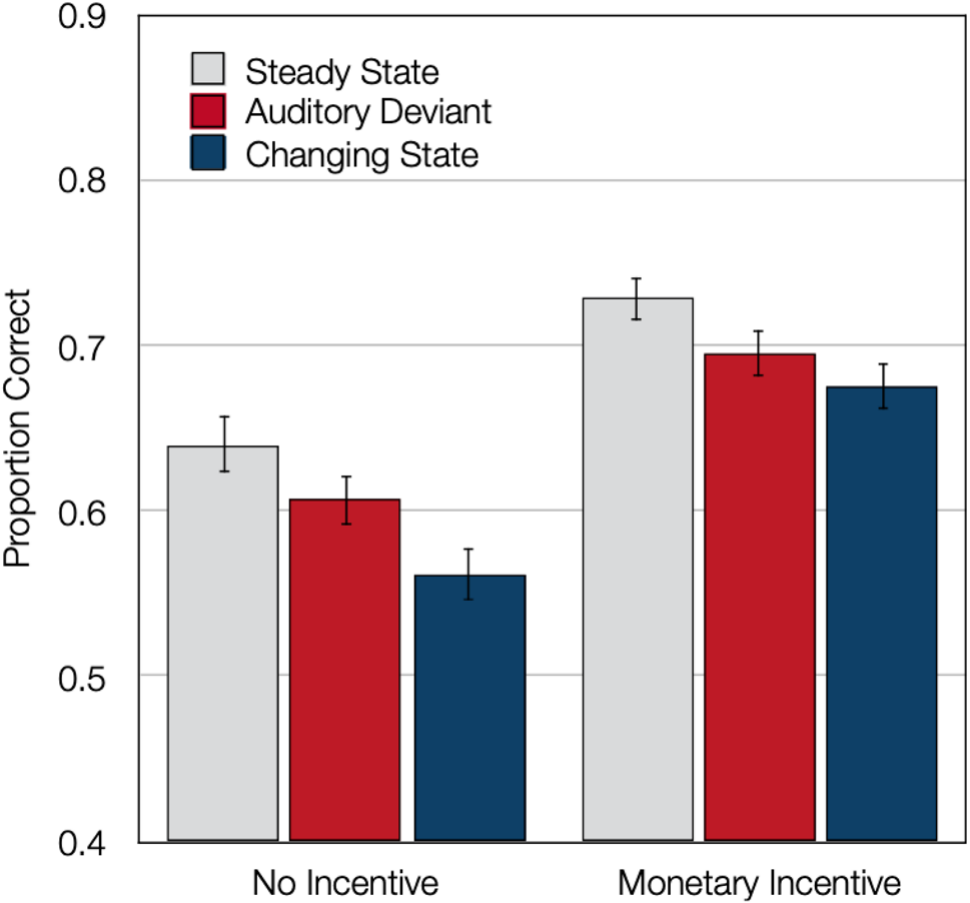
Results of Experiment 1. Serial recall in terms of proportion correct as a function of incentive and distractor condition. The error bars represent the standard errors of the means

As a manipulation check, we tested whether the presence or absence of monetary incentives affected performance in the control condition. As is evident from Fig. 1, the incentive variable had a pronounced effect on performance in the steady-state control condition, *F*(1,112) = 43.67, *p* < 0.001, η_p_^2^ = 0.28, suggesting that the manipulation of task engagement was successful.

In two supplementary analyses, the auditory-deviant effect and the changing-state effect were analyzed separately from each other. When the steady-state condition was contrasted with the auditory-deviant condition, there was evidence of an auditory-deviant effect, *F*(1,112) = 24.16, *p* < 0.001, η_p_^2^ = 0.18, but the auditory-deviant effect did not differ as a function of the levels of the incentive variable, *F*(1,112) < 0.01, *p* = 0.98, η_p_^2^ < 0.01. When the steady-state condition was contrasted with the changing-state condition, there was evidence of a changing-state effect, *F*(1,112) = 73.23, *p* < 0.001, η_p_^2^ = 0.40, which did not differ as a function of the levels of the incentive variable either, *F*(1,112) = 3.56, *p* = 0.06, η_p_^2^ = 0.03. Numerically there was a trend towards a slight reduction of the changing-state effect when performance was incentivized.

### Discussion

The presence or absence of monetary incentives substantially affected serial-recall performance in all of the distractor conditions. This finding shows that the monetary incentives were effective in inducing changes in task engagement. However, these changes in task engagement did not affect the size of the auditory-deviant effect. This was true even though the effect size of the auditory-deviant effect observed here (η_p_^2^ = 0.18) is in the order of magnitude of the effect sizes of the auditory-deviant effects observed in previous studies (η_p_^2^ = 0.23, 0.05, 0.17, and 0.18 in Experiments 1, 2, 3, and 4, of Hughes et al., 2005), which suggests that the auditory-deviant effect was faithfully reproduced. This pattern of findings supports the automatic-capture account according to which only the deliberate memorization of the target items is under cognitive control but attention capture by auditory deviants is not affected by cognitive control. According to this account, attention capture is based on automatic, stimulus-driven processes rather than on controlled attentional orienting. Numerically, there was a slight trend towards a reduction of the changing-state effect when performance was incentivized but the interaction was non-significant despite the comparatively large sample size. The present findings thus suggest that both the changing-state effect and the auditory-deviant effect can be classified as automatic because distraction persists even when participants are highly engaged in the focal task.

## Experiment 2

While Experiment 1 focused on stimulus-aspecific attentional diversion and thus on the first class of attentional diversion postulated by the duplex-mechanism model (Hughes, 2014), Experiment 2 focuses on the second class. Stimulus-specific attentional diversion is assumed to occur when the specific content of the auditory distractors captures the participants’ interest and leads them to shift their attention away from the memorization task. Specifically, the duplex-mechanism account includes the assumption that “any or some combination of the properties of a natural sentence that are absent from a single repeating word [causes] an additional, functionally distinct, attentional diversion effect over and above an underlying (pure) changing-state effect” (Hughes & Marsh, 2020, p. 430). According to this account, the stimulus-specific “attentional-diversion portion of the sentence effect […] is, like other attentional diversion effects […] attenuated by top-down factors” (p. 437). This leads to the prediction that the increased disruptive effect of sentential speech relative to that of a sequence of repeated one-syllable words should be modulated by incentive-induced changes in task engagement.

Testing whether external incentives may modulate the disruptive effect of naturalistic distractor speech is also interesting because results of a recent study were interpreted as providing hints of such a modulation. Ball et al. (2018) examined the effect of irrelevant speech on verbal problem solving in the compound remote-associates task. The influence of an external incentive was tested by promising participants a 4 GB USB pen drive dependent on their performance. In their Experiment 2, participants in a low-incentive condition were told that they had to reach at least 20% correct solutions while participants in a high-incentive condition had to reach at least 80% correct solutions to receive the bonus prize. In reality, all of the participants received the bonus prize at the end of the experiment regardless of their performance on the task. The interaction between incentive condition and distractor condition just missed the conventional level of statistical significance (*p* = 0.07), but the authors attached greater importance to the fact that the effect of irrelevant speech compared to quiet was numerically reduced (and no longer statistically significant) when performance was incentivized.

The present Experiment 2 can be seen as a follow-up to the study of Ball et al. (2018), but there are important methodological differences. (1) In the present study, we rely on the standard serial-recall paradigm to measure task performance. To anticipate, the effect of natural background speech on performance (relative to quiet) is much more pronounced in the present Experiment 2 (η_p_^2^ = 0.62) than in the study by Ball et al. (2018, η_p_^2^ = 0.09), which may suggest that the serial-recall task provides a particularly sensitive measurement of the effects of auditory distraction. (2) To test whether the disruptive effect of sentential speech is modulated by task engagement, we rely on the critical interaction between the incentive condition and the distractor condition as a decision criterion for the hypothesis test rather than on the presence or absence of the distraction effect at each level of the incentive variable; this is important because “the difference between ‘significant’ and ‘not significant’ is not itself statistically significant” (Gelman & Stern, 2006). (3) We use an even larger sample size in Experiment 2 (*N* = 173) than Ball et al. (2018, *N* = 68) to increase the sensitivity of Experiment 2 while maintaining the statistical power at the level of Experiment 1 (1 − β = 0.95). (4) We also use the more efficient within-subjects design already used in the present Experiment 1 rather than the less efficient between-subjects design used by Ball et al. (2018). In fact, pilot data obtained in our lab suggested that the effects of monetary incentives could not be reliably obtained with moderate sample sizes when the presence or absence of monetary incentives was manipulated between subjects. This is already evident from the fact that more than 350 participants would be needed to replicate an external incentive effect of the same size (η_p_^2^ = 0.035) as that observed in the study by Ball et al. (2018) with the conventional level of *α* = 0.05 and with sufficient statistical power (1 – β = 0.95) in a between-subjects design. The within-subjects design has the additional advantage that the prospect of monetary rewards may be more salient when the presence or absence of rewards changes from trial to trial so that trials with incentives are directly contrasted to trials without incentives. To anticipate, we were able to obtain a pronounced main effect of the incentive variable, which shows that task engagement was successfully manipulated. These conditions can be considered favorable for obtaining an interaction between incentive and auditory distraction if one exists.

An important reason for following up on the previous study of Ball et al. (2018) is that their study involved only the comparison of an irrelevant speech condition against a quiet control condition. However, the duplex-mechanism account specifically implies that the enhanced disruptive effect of sentential speech relative to that of a sequence of identical one-syllable words should be reduced by task engagement because the attentional diversion by sentential speech should be significantly reduced or even eliminated by top-down control. To test this prediction, the present Experiment 2 included not only a condition with sentential speech and a quiet control condition—as the study of Ball et al. (2018)—but also a steady-state condition with sequences of identical one-syllable words as distractors. This allowed us to test the prediction of the duplex-mechanism account that the effect of external incentives specifically reduces the difference in the disruptive potential of sentential speech versus that of sequences of one-syllable words (Hughes & Marsh, 2020, p. 430).

### Method

#### Participants

We aimed at recruiting as many participants as possible in the two weeks the laboratory was available to us. During that time, we collected valid data sets of 173 participants (135 of whom were female). One additional participant did not finish the experiment, as a consequence of which no data file was saved. Given *α * = 0.05 and the assumption that the population correlation of the differences between the no-incentive and the monetary-incentive condition among the levels of the distractor condition variable is *ρ* = 0.5, a total sample size of *N* = 173 allowed us to detect an interaction effect of the size η_p_^2^ = 0.08 with a statistical power of 1 – β = 0.95. All participants were recruited on campus of Heinrich Heine University Düsseldorf. Their age ranged from 17 to 38 years with a mean age of 23 (*SD* = 4) years. The participants received course credit or a monetary compensation of 4 € for participating. In addition, they could earn up to 4.68 € depending on their performance in the serial-recall task.

#### Design, materials and procedure

A 2 × 3 design was used with incentive (no incentive, monetary incentive) and distractor condition (quiet, steady state, sentential speech) as repeated-measures variables and serial recall as a dependent variable.

Materials and procedure were identical to those of Experiment 1 with the following exceptions. To familiarize participants with the procedure, the experiment started with four quiet training trials. Then 16 quiet trials, 16 steady-state trials, and 16 sentential-speech trials followed in a randomly determined order that was individually generated for each participant.

The same sentential-speech and steady-state distractor material were used as in previous studies (e.g., Röer et al., 2015). In the sentential-speech condition, sentences were played (e.g., “Peel and quarter the onions and slice them into thin pieces, then add the tomatoes, then simmer at medium heat”; translated from German) that were taken from eight different categories (weather forecast, prose text, cooking recipe, scientific textbook, poem, operating manual, road message, aphorism). For each steady-state sequence, a monosyllabic word (from one of the sentences) was randomly selected and repeated 18 times, the latter of which corresponded to the mean number of words in the changing-state sequences. The auditory sequences were spoken by a male voice and lasted 8 s each. On average, the experiment lasted about 26 min and participants earned 3.22 € based on their performance in the incentivized trials.

### Results

There was a main effect of distractor condition, *F*(2,171) = 140.11, *p* < 0.001, η_p_^2^ = 0.62. As in Experiment 1, serial recall was better when good performance was incentivized than when it was not, *F*(1,172) = 114.94, *p* < 0.001, η_p_^2^ = 0.40. Other than in Experiment 1, there was also a significant interaction between the incentive and the distractor condition variables, *F*(2,171) = 4.26, *p* = 0.02, η_p_^2^ = 0.05, suggesting that auditory distraction was affected by the monetary incentives. However, there was still a pronounced and statistically significant effect of distraction on serial recall when performance was incentivized, *F*(2,171) = 78.84, *p* < 0.001, η_p_^2^ = 0.48. Distraction was thus only slightly reduced in the monetary-incentive condition and not completely abolished.

**Fig. 2:**
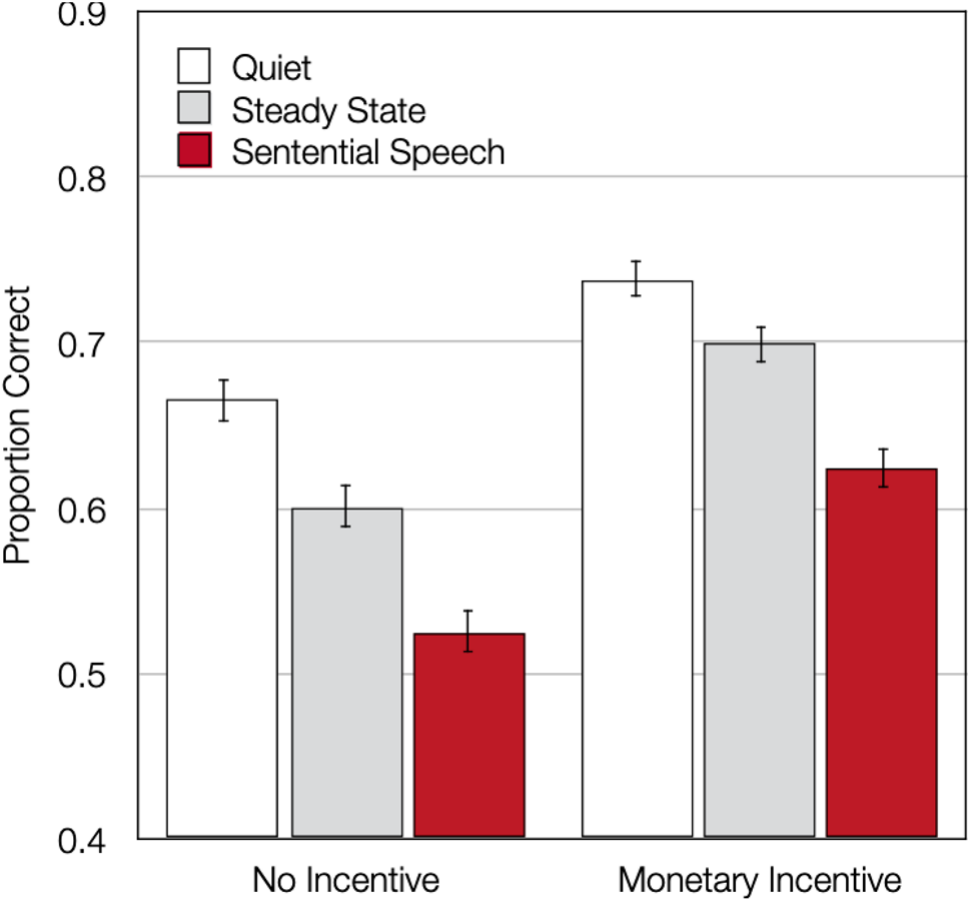
Results of Experiment 2. Serial recall in terms of proportion correct as a function of incentive and distractor condition. The error bars represent the standard errors of the means

As a manipulation check, we tested whether the presence or absence of monetary incentives affected performance in the quiet control condition. As is evident from Fig. 2, the incentive variable had a pronounced effect on performance in the quiet control condition, *F*(1,172) = 56.76, *p* < 0.001, η_p_^2^ = 0.25, suggesting that the manipulation of task engagement was successful.

As the next step, we tested the prediction of the duplex-mechanism account that task engagement should specifically affect the disruptive effect of sentential speech relative to that of simple steady-state sequences of one-syllable words (Hughes & Marsh, 2020). Contrary to this prediction, only the steady-state effect (that is, the disruptive effect of steady-state sounds relative to the quiet control condition) was affected by the incentives. Specifically, when the steady-state condition was contrasted with the quiet condition, a significant effect of distraction was obtained, confirming that steady-state speech significantly disrupted performance relative to quiet, *F*(1,172) = 79.03, *p* < 0.001, η_p_^2^ = 0.31. This steady-state effect was significantly modulated by the presence or absence of incentives, *F*(1,172) = 7.00, *p* = 0.01, η_p_^2^ = 0.04. By contrast, when the sentential-speech condition was contrasted with the steady-state condition, there was clear evidence of an increased disruption of serial recall by sentential speech relative to steady-state speech, *F*(1,172) = 139.83, *p* < 0.001, η_p_^2^ = 0.45, but the difference in the disruptive potential of sentential speech compared to steady-state speech was essentially the same regardless of whether good performance was rewarded or not, *F*(1,172) < 0.01, *p* = 0.96, η_p_^2^ < 0.01.

### Discussion

In line with Experiment 1, the monetary incentives had a strong effect on baseline performance. This finding demonstrates that the presence or absence of monetary rewards was effective in inducing changes in task engagement, which affected the controlled processes underlying the memorization of the target items. In line with the conclusions of Ball et al. (2018), the disruptive effect of irrelevant speech compared to a quiet control condition was reduced when external incentives for good task performance were provided. However, in contrast to that previous study, the effect of distraction on performance was only slightly reduced by the incentive-induced increase in task engagement and thus remained significant even when good performance was rewarded. This finding cannot be explained by a failure of the incentives to affect task engagement given that there was a pronounced effect of the presence of incentives on performance in all conditions. Instead, the difference between studies is likely due to the fact that the effect of auditory distraction on serial recall was generally stronger than that on verbal problem solving so that it was less likely to fall below the level of statistical significance under conditions of high task engagement.

The main purpose of Experiment 2 was to test the prediction of the duplex-mechanism account of auditory distraction that incentive-induced changes in task engagement should selectively affect the disruptive effect of sentential speech relative to that of a steady-state condition. This hypothesis is derived from the assumption that the greater disruptive effect of sentential speech relative to sequences of one-syllable words is partly caused by attentional diversion, and that the “meaning and hence ‘relevance’or ‘interest’ of a sentence is key to its attention-diverting power” (Hughes & Marsh, 2020, p. 438). Importantly, the duplex-mechanism account implies that the orienting of attention towards the meaning of a sentence is under cognitive control and should therefore be abolished under conditions of high task engagement. The present data provide evidence against this hypothesis as the enhanced disruptive effect of sentential speech relative to that of steady-state speech remained completely unaffected by the incentive-induced manipulation of task engagement. This provides evidence that people have only limited cognitive control over the distraction by sentential speech.

Experiment 2 also allowed us to compare the disruptive effect of steady-state speech relative to a quiet condition. According to the duplex-mechanism account, steady-state sequences should produce little to no disruption (Hughes, 2014) because they are highly predictable and contain no order information. However, a review of the available literature as well as new empirical evidence (Bell et al., 2019b) has recently shown that evidence for a disruptive effect of steady-state speech relative to quiet is robustly obtained in adequately powered studies (with sample sizes of *N* > 40). The present study confirms this conclusion as steady-state speech caused a significant decrease in performance relative to the quiet control condition. The sample effect size (η_p_^2^ = 0.31) lies between those obtained for the changing-state effect (η_p_^2^ = 0.40) and the auditory-deviant effect (η_p_^2^ = 0.18) in Experiment 1. Interestingly, the size of the steady-state effect was significantly modulated by changes in task engagement, suggesting that people have some capacity to suppress auditory distraction relative to a quiet control condition when they are highly engaged in a task. This modulation of the steady-state effect by task engagement is interesting and may inform future theoretical explanations of the effect, but one should be cautious about interpreting this finding at present for two reasons. First, none of the models considered here makes strong predictions about how this effect should be modulated. Second, the modulation is small in terms of its sample effect size. However, if the modulation of the steady-state effect by task engagement turns out to be replicable in future studies, this may indicate that top-down control affects cross-modal distraction at early stages of processing when—or even before—a call for attention is elicited, consistent with the notion that auditory distractors in cross-modal paradigms can be filtered out already at a subcortical level (Guerreiro et al., 2010). In any case, it is important to note that distraction is only slightly modulated but not completely abolished since strong distraction effects were observed regardless of the level of task engagement.

## General discussion

The main purpose of the present study is to test the theoretically derived hypothesis that stimulus-aspecific attentional diversion (Experiment 1) and stimulus-specific attentional diversion (Experiment 2) by auditory distractors are under cognitive control and should therefore be affected by incentive-induced changes in task engagement. The present results contradict the view that attentional orienting to auditory distractors is under cognitive control. Both stimulus-aspecific attentional diversion—exemplified by the auditory-deviant effect (Hughes et al., 2013)—and stimulus-specific attentional diversion—exemplified by the increased disruptive potential of sentential speech relative to that of sequences of one-syllable words (Hughes & Marsh, 2020)—remained unaffected by incentive-induced changes in task engagement, just as the changing-state effect observed in Experiment 1.^1^ In line with the conclusions drawn from a recent study on verbal problem solving (Ball et al., 2018), external incentives caused a numerically small but statistically significant reduction of auditory distraction relative to quiet in Experiment 2. However, in contrast to that previous study, auditory distraction was only slightly reduced and not completely abolished. Strong effects of auditory distraction on performance persisted even in states of high task engagement in both experiments reported here.

The present results are thus broadly consistent with the automatic-capture account, according to which attentional orienting to auditory distractors is largely underpinned by the automatic perceptual analysis of the auditory input and occurs despite the individual’s best effort to concentrate on the memorization task (e.g., Körner et al., 2017; Röer et al., 2017). According to this account, both the attention capture by auditory deviants and the processing of naturalistic distractor speech is largely automatic which leaves only the processes responsible for the deliberate memorization of the target items as being primarily dependent on controlled mental effort. Accordingly, the incentive-induced changes in task engagement primarily caused a global change in serial-recall performance but did not substantially affect attention capture by auditory distractors. These findings are consistent with the understanding of attention capture as a largely stimulus-driven response to deviating or novel stimuli that is prevalent in related fields of research (e.g., Parmentier et al., 2008; Parmentier & Gallego, 2020). While a lack of top-down control over auditory distraction may, at first glance, appear to be a flaw, it is often seen as a necessary cost for maintaining the delicate balance between the conflicting goals of openness and selectivity (e.g., Cowan, 1995; Escera et al., 2000; Schröger, 1996). This functional view implies that the attentional system is likely not designed to deal with situations in which the auditory modality can be ignored in its entirety for the simple reason that such situations do not exist. Outside of the laboratory, whether perceptual input is relevant or irrelevant is rarely as clearly defined as it *nominally* is in selective-attention paradigms. Auditory signals have to be processed to some degree even when they are not directly relevant for an ongoing task because being able to respond to auditory signals such as auditory alarms or human speech-based communication signals is crucial for survival even when concentrating on an unrelated task. Abrupt changes in the auditory modality and violations of auditory regularities, in particular, signal potentially important changes in the environment and thus are associated with increased demands of processing before their relevance or irrelevance for the individual’s goals can be determined. The processing of changing and deviating distractors is thus to some degree mandatory and should not be completely abolished by increased task engagement.

Given that the main conclusion from the present study is that changes in task engagement have only limited effects on attentional diversion by auditory distractors, it is important that the analysis of the serial-recall performance shows that the manipulation of task engagement was successful. In previous studies (Hughes et al., 2013; Marsh et al., 2018, 2020), the proof of the success of the task-engagement manipulations solely relied on the fact that it was effective in reducing auditory distraction. As a criterion for determining the success of the task-engagement manipulation, this is not ideal because this approach does not allow to falsify the hypothesis that enhanced task engagement affects distraction. Self reports are not ideal either as they may be strongly biased by salient cues and social desirability. Therefore, we proposed here to rely on performance in the control condition as a simple yet direct and clear-cut test of the effectiveness of the task-engagement manipulation. According to this criterion, the incentive-induced manipulation of task engagement was successful as the provision of monetary rewards led to an increase of roughly 14% in performance in the steady-state control condition in Experiment 1 and of roughly 11% in the quiet control condition in Experiment 2. The incentive effect was even larger—both in terms of the relative change in performance and in terms of effect size—than benchmarks of working memory (Oberauer et al., 2018) such as the auditory-deviant effect and the changing-state effect.

Given that it has been previously demonstrated that distraction by auditory deviants (Hughes et al., 2013; Marsh et al., 2018) and sentential speech (Hughes & Marsh, 2020; Röer et al., 2015) can be abolished or at least reduced by manipulations thought to reflect cognitive control, the question arises how this inconsistency can be resolved. It is first worth pointing out that dissociations between factors that are grouped together under the label of cognitive control in the duplex-mechanism account are common in many other areas of research (e.g., Parmentier, 2014; Parmentier et al., 2008). For instance, stimulus-driven competition effects under high perceptual load have often found to be dissociated from top-down cognitive control (Lavie et al., 2004). From the perspective of the duplex-mechanism account (Hughes, 2014), these dissociations are highly surprising because they do not fit the postulated dichotomy between controlled and automatic processing. However, it has long been realized that empirical dissociations do not provide conclusive evidence for fundamental dichotomies in cognition: Dissociations are the rule rather than the exception even when using measures that tap into the same construct (e.g., Kolers & Roediger, 1984). Empirical dissociations may be produced by simple methodological artifacts (e.g., Buchner & Wippich, 2000) or by any of the multiple component processes involved in empirical tests (Hintzman, 1990) and do not conclusively prove the existence of two fundamentally different types of processing (Keren & Schul, 2009). This ambiguity complicates the interpretation of empirical dissociations. In the next paragraph, this problem is illustrated using the effects of visual warnings on auditory distraction as an example.

In previous studies, it has been shown that visual warnings informing about the type of distractor that had to be ignored in the following trial had a selective effect on performance in the sense that it benefitted performance more when the warning was about auditory deviants (Hughes et al., 2013; but see Bell et al., 2017) or sentential speech (Bell et al., 2017; Hughes & Marsh, 2020; Röer et al., 2015) than when it was about changing-state or steady-state speech. In the present study, by contrast, the visual warning indicating the presence of rewards had a global effect on performance. At first glance, these findings appear to be contradictory because they do not fit the postulated dichotomy of controlled and automatic processes. While these results seem puzzling from the perspective of the duplex-mechanism account (Hughes, 2014), they can be resolved by realizing that the effectiveness of a visual warning does not only depend on the amenability of distraction to cognitive control but also on the nature of the warning itself. In the previous studies, the forewarnings always predicted the upcoming distractor truthfully which implies that the type of distractor speech that had to be ignored during the trial was inherently confounded with the type of warning presented before the trial. To illustrate, in one study the warning “Deviant” flashed on and off in red font before auditory-deviant trials while the warning “No Deviant” was continuously displayed in black font before trials in which no deviant had to be ignored (Hughes et al., 2013, Experiment 2). To the degree that these warnings are differentially effective in communicating the need to invest mental effort in the task, selective effects on performance are to be expected based on the present results. Direct evidence for the importance of the quality of the warnings comes from a study by Röer et al. (2015) in which only specific foreknowledge about the content of an upcoming distractor sentence significantly reduced distraction by the spoken sentence while the mere notification about the imminent presentation of an unspecified sentence had no effect on performance. Manipulations that help participants to predict the upcoming distraction thus seem particularly effective in reducing the disruptive effect of sentential speech. This example demonstrates that empirical dissociations are easily obtained even when the same type of distractor material is used and thus are of limited diagnostic value for distinguishing between two fundamentally different types of processing modes.

Within the duplex-mechanism account, empirical dissociations interpreted as being conditional upon different types of distractors are seen as diagnostic of two different forms of processing modes while findings that do not fit into this dichotomy are dismissed as resulting from methodological artifacts (e.g., Hughes & Marsh, 2020). However, it is also possible to argue that the overall data pattern does not support the existence of two coherent, functionally dissociable forms of auditory distraction. The objective of this criticism is not to negate that different types of distractors have different properties that may be associated with different processing requirements and outcomes, but to emphasize that the available body of evidence arguably does not map well onto two functionally isolable, non-overlapping forms of auditory distraction that are each governed by dissociated sets of correlated characteristics. Specifically, the present results suggest that the characterization of attentional diversion as a type of behavior that is under cognitive control—akin to “Type II” processing (Hughes, 2014, p. 31) in dual-process theories (Keren & Schul, 2009)—misrepresents the nature of the underlying mechanism.

From an applied point of view, it is interesting that external incentives are effective in modulating global task engagement but at the same time are of limited use to specifically counter auditory distraction. While salient changes in monetary rewards can affect how much mental effort people are willing to put into a task and may thereby influence performance in tasks that rely on controlled processing and working memory (see also Morey et al., 2011), the presence of monetary rewards does not eliminate the detrimental performance effects of auditory distractors that arguably capture attention in a primarily stimulus-driven manner. It thus seems important to implement effective noise abatement strategies to protect cognitive processing from distraction in real-world settings where good cognitive performance is important because real incentives are at stake.

## Footnotes

1 As an alternative to the Neyman-Pearson approach used here, a reviewer suggested to perform a Bayesian analysis to quantify the evidence for the null hypothesis. The Bayesian analysis can be found at: https://osf.io/m68y5/.

## References

[CR1] Ball LJ, Threadgold E, Solowiej A, Marsh JE (2018). Can intrinsic and extrinsic metacognitive cues shield against distraction in problem solving?. Journal of Cognition.

[CR2] Banbury SP, Macken WJ, Tremblay S, Jones DM (2001). Auditory distraction and short-term memory: Phenomena and practical implications. Human Factors.

[CR3] Bell R, Mieth L, Röer JP, Troche SJ, Buchner A (2019). Preregistered replication of the auditory deviant effect: A robust benchmark finding. Journal of Cognition.

[CR4] Bell R, Röer JP, Lang A-G, Buchner A (2019). Distraction by steady-state sounds: Evidence for a graded attentional model of auditory distraction. Journal of Experimental Psychology: Human Perception and Performance.

[CR5] Bell R, Röer JP, Lang A-G, Buchner A (2019). Reassessing the token set size effect on serial recall: Implications for theories of auditory distraction. Journal of Experimental Psychology: Learning, Memory, and Cognition.

[CR6] Bell R, Röer JP, Marsh JE, Storch D, Buchner A (2017). The effect of cognitive control on different types of auditory distraction: A preregistered study. Experimental Psychology.

[CR7] Bredenkamp J, Erdfelder E (1985). Multivariate Varianzanalyse nach dem V-Kriterium [Multivariate analysis of variance based on the V-criterion]. Psychologische Beitrage.

[CR8] Buchner A, Wippich W (2000). On the reliability of implicit and explicit memory measures. Cognitive Psychology.

[CR9] Bucker B, Theeuwes J (2014). The effect of reward on orienting and reorienting in exogenous cuing. Cognitive, Affective and Behavioral Neuroscience.

[CR10] Campbell T, Beaman CP, Berry DC (2002). Auditory memory and the irrelevant sound effect: Further evidence for changing-state disruption. Memory.

[CR11] Cowan N (1995). Attention and memory: An integrated framework. Oxford University Press.

[CR12] Ellermeier W, Zimmer K (2014). The psychoacoustics of the irrelevant sound effect. Acoustical Science and Technology.

[CR13] Escera C, Alho K, Schröger E, Winkler I (2000). Involuntary attention and distractibility as evaluated with event-related brain potentials. Audiology and Neuro Otology.

[CR14] Gelman A, Stern H (2006). The difference between “significant” and “not significant” is not itself statistically significant. The American Statistician.

[CR15] Guerreiro MJS, Murphy DR, Van Gerven PWM (2010). The role of sensory modality in age-related distraction: A critical review and a renewed view. Psychological Bulletin.

[CR16] Hintzman DL (1990). Human learning and memory: Connections and dissociations. Annual Review of Psychology.

[CR17] Hughes RW (2014). Auditory distraction: A duplex-mechanism account. PsyCH.

[CR18] Hughes RW, Hurlstone MJ, Marsh JE, Vachon F, Jones DM (2013). Cognitive control of auditory distraction: Impact of task difficulty, foreknowledge, and working memory capacity supports duplex-mechanism account. Journal of Experimental Psychology: Human Perception and Performance.

[CR19] Hughes RW, Marsh JE (2020). When is forewarned forearmed? Predicting auditory distraction in short-term memory. Journal of Experimental Psychology: Learning Memory and Cognition.

[CR20] Hughes RW, Vachon F, Jones DM (2005). Auditory attentional capture during serial recall: Violations at encoding of an algorithm-based neural model?. Journal of Experimental Psychology: Learning, Memory, and Cognition.

[CR21] Hughes RW, Vachon F, Jones DM (2007). Disruption of short-term memory by changing and deviant sounds: Support for a duplex-mechanism account of auditory distraction. Journal of Experimental Psychology: Learning, Memory, and Cognition.

[CR22] Jones DM, Macken WJ (1993). Irrelevant tones produce an irrelevant speech effect: Implications for phonological coding in working memory. Journal of Experimental Psychology: Learning, Memory, and Cognition.

[CR23] Jones DM, Macken WJ, Murray AC (1993). Disruption of visual short-term memory by changing-state auditory stimuli: The role of segmentation. Memory & Cognition.

[CR24] Keren G, Schul Y (2009). Two is not always better than one. Perspectives on Psychological Science.

[CR25] Kolers PA, Roediger HL, III.  (1984). Procedures of mind. Journal of Verbal Learning & Verbal Behavior.

[CR26] Körner U, Röer JP, Buchner A, Bell R (2017). Working memory capacity is equally unrelated to auditory distraction by changing-state and deviant sounds. Journal of Memory and Language.

[CR27] Lange E (2005). Disruption of attention by irrelevant stimuli in serial recall. Journal of Memory and Language.

[CR28] Lavie N, Hirst A, De Fockert JW, Viding E (2004). Load theory of selective attention and cognitive control. Journal of Experimental Psychology: General.

[CR29] LeCompte DC, Neely CB, Wilson JR (1997). Irrelevant speech and irrelevant tones: The relative importance of speech to the irrelevant speech effect. Journal of Experimental Psychology: Learning, Memory, and Cognition.

[CR30] Marsh JE, Campbell TA, Vachon F, Taylor PJ, Hughes RW (2020). How the deployment of visual attention modulates auditory distraction. Attention, Perception, & Psychophysics..

[CR31] Marsh JE, Röer JP, Bell R, Buchner A (2014). Predictability and distraction: Does the neural model represent post-categorical features?. PsyCH.

[CR32] Marsh JE, Yang J, Qualter P, Richardson C, Perham N, Vachon F, Hughes RW (2018). Post-categorical auditory distraction in serial short-term memory: Insights from increased task load and task type. Journal of Experimental Psychology: Learning, Memory, and Cognition.

[CR33] Morey CC, Cowan N, Morey RD, Rouder JN (2011). Flexible attention allocation to visual and auditory working memory tasks: manipulating reward induces a trade-off. Attention, Perception, & Psychophysics.

[CR34] O'Brien RG, Kaiser MK (1985). MANOVA method for analyzing repeated measures designs: An extensive primer. Psychological Bulletin.

[CR35] Oberauer K, Lewandowsky S, Awh E, Brown GDA, Conway ARA, Cowan N, Donkin C, Farrell S, Hitch GJ, Hurlstone MJ, Ma WJ, Morey CC, Nee DE, Schweppe J, Vergauwe E, Ward G (2018). Benchmarks for Models of Short Term and Working Memory. Psychological Bulletin.

[CR36] Parmentier FBR (2014). The cognitive determinants of behavioral distraction by deviant auditory stimuli: A review. Psychological Research Psychologische Forschung.

[CR37] Parmentier FBR, Elford G, Escera C, Andres P, San Miguel I (2008). The cognitive locus of distraction by acoustic novelty in the cross-modal oddball task. Cognition.

[CR38] Parmentier FBR, Gallego L (2020). Is deviance distraction immune to the prior sequential learning of stimuli and responses?. Psychonomic Bulletin & Review.

[CR39] Pochon JB, Levy R, Fossati P, Lehericy S, Poline JB, Pillon B, Le Bihan D, Dubois B (2002). The neural system that bridges reward and cognition in humans: An fMRI study. Proceedings of the National Academy of Sciences.

[CR40] Röer JP, Bell R, Buchner A (2014). What determines auditory distraction? On the roles of local auditory changes and expectation violations. PLoS ONE.

[CR41] Röer JP, Bell R, Buchner A (2015). Specific foreknowledge reduces auditory distraction by irrelevant speech. Journal of Experimental Psychology: Human Perception and Performance.

[CR42] Röer JP, Körner U, Buchner A, Bell R (2017). Attentional capture by taboo words: A functional view of auditory distraction. Emotion.

[CR43] Schlittmeier SJ, Hellbrück J, Klatte M (2008). Does irrelevant music cause an irrelevant sound effect for auditory items?. European Journal of Cognitive Psychology.

[CR44] Schröger E (1996). A neural mechanism for involuntary attention shifts to changes in auditory stimulation. Journal of Cognitive Neuroscience.

[CR45] Shenhav A, Botvinick MM, Cohen JD (2013). The expected value of control: An integrative theory of anterior cingulate cortex function. Neuron.

[CR46] Shenhav A, Cohen JD, Botvinick MM (2016). Dorsal anterior cingulate cortex and the value of control. Nature Neuroscience.

[CR47] Sörqvist P (2010). High working memory capacity attenuates the deviation effect but not the changing-state effect: Further support for the duplex-mechanism account of auditory distraction. Memory & Cognition.

[CR48] Taylor SF, Welsh RC, Wager TD, Phan KL, Fitzgerald KD, Gehring WJ (2004). A functional neuroimaging study of motivation and executive function. NeuroImage.

[CR49] Tremblay S, Jones DM (1999). Change of intensity fails to produce an irrelevant sound effect: Implications for the representation of unattended sound. Journal of Experimental Psychology: Human Perception and Performance.

[CR50] Vachon F, Labonté K, Marsh JE (2017). Attentional capture by deviant sounds: A noncontingent form of auditory distraction?. Journal of Experimental Psychology: Learning, Memory, and Cognition.

[CR51] Walsh AT, Carmel D, Harper D, Grimshaw GM (2018). Motivation enhances control of positive and negative emotional distractions. Psychonomic Bulletin & Review.

[CR52] Wiens S, Szychowska M, Nilsson ME (2016). Visual task demands and the auditory mismatch negativity: An empirical study and a meta-analysis. PLoS ONE.

[CR53] Wiens S, van Berlekom E, Szychowska M, Eklund R (2019). Visual perceptual load does not affect the frequency mismatch negativity. Frontiers in Psychology.

